# Grading the strength and certainty of the scientific evidence of the bidirectional association between periodontitis and noncommunicable diseases: an umbrella review

**DOI:** 10.1038/s41432-025-01132-9

**Published:** 2025-03-13

**Authors:** Carolina Rodríguez-Medina, Sandra Amaya Sánchez, Adolfo Contreras, Javier Enrique Botero

**Affiliations:** 1 Facultad de Odontología, Universidad de Antioquia, Calle 70# 52-21, Medellín, Colombia; 2 Escuela de Odontología, Universidad del Valle, Cali, Colombia

**Keywords:** Periodontitis, Dental public health, Occupational health

## Abstract

**Objective:**

Periodontitis and various noncommunicable diseases (NCDs) have been proposed to have a bidirectional relationship. The purpose of this umbrella review is (1) to synthesize the evidence and (2) to grade the strength and certainty of the scientific evidence regarding the bidirectional association between periodontitis and NCDs.

**Data sources:**

Electronic databases were systematically searched from January 2021 and July 2024; MEDLINE (via PubMed), Embase and SciELO.

**Data selection and extraction:**

Potential epidemiologic systematic reviews with meta-analysis that studied the bidirectional association between periodontitis and NCDs were identified by two independent reviewers and filtered by title and abstract according to the selection criteria. The strength and the quality and certainty of the evidence was assessed according to the Grading of Recommendations, Assessment, Development and Evaluations (GRADE) guide. 561,554 potential results were identified. After removing duplicates and excluding records deemed ineligible by automated filters, 450 results were screened by title and abstract. This process led to 41 records being appraised in full-text. Of these, 17 were further excluded leaving a total of 24 systematic reviews that met the inclusion criteria.

**Data synthesis:**

24 systematic reviews with a total of 32 NCDs were appraised and consolidated. Risk of bias assessment indicated that 21 systematic reviews (87.5%) demonstrated low bias (high quality), 2 had medium bias, and 1 exhibited high bias (low quality). Key issues identified included the formulation of explicit research questions, critical appraisal, data extraction, and publication bias. The association between periodontitis and NCDs was strong in 1 systematic review, moderate in 8, weak in 10 and absent in 7 systematic reviews. The strength of the association between NCDs and periodontitis was moderate in 6 systematic reviews and weak in 3 systematic reviews. The size of the reported effect (odds ratio/risk ratio/hazard ratio) was broader with increasing strength. Although data supports the association between periodontitis and some NCDs, and to a lesser extent between some NCDs and periodontitis, the certainty of the evidence was classified as low to very low.

**Conclusions:**

There is some data that, with varying degrees of association and low to very low certainty, provide evidence that periodontitis may be a potential risk factor for some NCDs and vice versa.

Key points

- Clinicians should be aware of the possible links between oral and systemic health but should interpret these associations with caution.
- The review revealed methodological issues in systematic reviews, such as publication bias, a lack of specific research goals and diversity in critical appraisal and data extraction procedures.
- When counseling patients, healthcare providers should avoid exaggerating the role of periodontitis in systemic disorders. Instead, they should stress the well-established benefits of periodontal care, including improved oral health and lower local inflammation, while remaining open to new findings.

## Introduction

Periodontitis is a chronic inflammatory disease primarily caused by a dysbiotic bacterial biofilm adhered to dental surfaces with the potential to exacerbate systemic inflammation, consequently leading to or worsening conditions such as cardiovascular diseases, diabetes, and respiratory diseases. The inflammatory mediators released during periodontal infections, such as cytokines and prostaglandins, can enter the bloodstream, contributing to systemic inflammation and promoting the development of systemic health complications [1, 2].

Noncommunicable diseases (NCDs) are medical conditions that cannot be transmitted directly from one person to another. They are usually chronic and progress slowly, frequently lasting for many years or even a lifetime. Cardiovascular complications, malignancies, chronic respiratory diseases, and diabetes are the four most common types of NCDs. These disorders are caused by a mix of genetic, physiological, environmental, and behavioral factors. Tobacco use, physical inactivity, a poor diet, and excessive alcohol consumption all contribute to metabolic alterations such as hypertension, obesity, hyperglycemia, and hyperlipidemia [3]. The impact of NCDs on human health is considerable, imposing a significant burden on individuals, healthcare systems, and economies around the world. NCDs are the main cause of mortality worldwide, accounting for over 74% of all deaths each year. They cause extended disability, lower quality of life, and higher healthcare costs. The chronic nature of NCDs involves long-term management and care, which places significant strain on healthcare resources. Furthermore, NCDs disproportionately impact low- and middle-income nations, where healthcare systems may be less suited to manage the complex and long-term care needed [3].

Periodontitis and some NCDs have a bidirectional relationship, meaning that the existence of one condition might influence the severity or progression of the other. This interaction is mostly explained by the systemic inflammatory response and immunological regulation. In 2016, Monsarrat and coworkers conducted a search related to periodontal medicine and reported that 57 systemic conditions have hypothetically associated with periodontitis. Apart from cardiovascular conditions, diabetes and adverse pregnancy outcomes which have abundant evidence, other diseases included anemia, liver diseases, dyspepsia and ankylosing spondylitis [4].

The complex relationship between periodontitis and NCDs emphasizes the significance of comprehensive healthcare treatments that address both oral and systemic health. Clinicians must acknowledge the interconnectivity of various NCDs and use a holistic approach to diagnose and treat the patients. For example, addressing periodontal disease may help manage systemic inflammation, positively impacting illnesses like cardiovascular disease and diabetes [5]. Likewise, effectively managing systemic conditions can contribute to better periodontal health, highlighting the need for collaborative care between dental and medical professionals. This integrated approach can lead to improved overall health outcomes and quality of life for patients.

The abundant publication of systematic reviews has resulted in an overwhelming collection of research that can be both repetitive and contradicting, which can cause scientific, ethical, economic, and social consequences [6]. While systematic reviews are useful for combining primary research, their methodology, quality, and scope can vary greatly, resulting in a range of results and recommendations. The large volume of these reviews can lead to information overload, making it challenging for physicians, policymakers, and academics to identify the most relevant and high-quality data needed for evidence-based decision-making.

Umbrella reviews address these challenges by providing a comprehensive synthesis of systematic reviews and meta-analyses on a particular topic. They offer a higher level of evidence by critically appraising and integrating findings from multiple systematic reviews, thus delivering a more cohesive and reliable summary of the existing research. Umbrella reviews enhance the clarity and utility of the evidence base, highlighting areas of consensus and identifying gaps or inconsistencies in the literature. Therefore, the purpose of this umbrella review is (1) to synthesize the evidence and (2) to grade the strength and certainty of the scientific evidence regarding the bidirectional association between periodontitis and NCDs.

## Methods

An umbrella review was conducted according to the recommendations of the JBI (Joanna Briggs Institute) and adhered to the Preferred Reporting Items for Systematic Reviews and Meta-Analyses (PRISMA) framework.

### Focused question

Two focused questions were formulated using the PECO strategy depending on the direction of the association.

First direction: periodontitis is a risk factor for NCDs.

**Table:** 

Population	(P):	adults.
Exposure	(E):	periodontitis.
Comparator	(C):	no periodontitis.
Outcome	(O):	frequency of periodontitis in individuals with and without NCDs.

What is the association between periodontitis and NCDs in adults?

Second direction: NCDs are a risk factor for periodontitis.

**Table:** 

Population	(P):	adults.
Exposure	(E):	NCDs.
Comparator	(C):	no NCDs.
Outcome	(O):	frequency of NCDs in individuals with and without periodontitis.

What is the association between NCDs and periodontitis in adults?

### Definition of NCDs

NCDs were defined as the most common systemic diseases that have been associated with periodontitis. To assist with the listing of possible NCDs, an artificial intelligence (AI-ChatGPT 4.0: searched July 24 2024) was used (see Supplementary Material 1). It is important to note that in some instances bidirectionality does not apply according to the current biological plausibility of the studied relationship and therefore was not included in the search.

### Inclusion criteria

Only systematic reviews with meta-analysis were included in the review. There was no language restriction, but reviews published in languages other than Spanish or English were translated using an application.

### Search strategy

A comprehensive search was conducted by two independent reviewers in MEDLINE (via PubMed), Embase and SciELO, restricted to a timeframe between January 2021 and July 2024, to retrieve the most recent evidence. The Cochrane Database of Systematic Reviews was not searched, as these reviews are included in PubMed and are typically limited to interventions. A list of keywords was used in various combinations (see Supplementary Material 2). The search was automatically restricted by time frame and type of article (i.e., systematic reviews).

### Inclusion of systematic reviews

Initially, potential epidemiologic systematic reviews with meta-analysis that studied the bidirectional association between periodontitis and NCDs were identified by two independent reviewers and filtered by title and abstract according to the selection criteria. Subsequently, systematic reviews were selected for full-text review based on these criteria. Full-text articles were then retrieved, reviewed, and included. Any discrepancies between the reviewers were discussed, and if consensus could not be reached, a third reviewer made the final determination on study inclusion. The PRISMA flow diagram was used to illustrate the study selection process.

If multiple systematic reviews were identified for a given disease (e.g., diabetes), only the most recent report that met the inclusion criteria at the time of assessment was included to avoid overlapping. If the most recent systematic review did not meet the inclusion criteria, the next most recent review was considered, and so on. Critically low quality reviews were excluded as well as any other type of review that did not meet the inclusion criteria (e.g. animal studies, in vitro studies). Therefore, a single systematic review with meta-analysis was included for a singular disease depending on the availability of the evidence.

### Data extraction

Data was extracted by two independent reviewers using a predetermined form that included: year and author, country of origin, systemic disease, number of studies, number of high risk of bias, number of events and totals in cases and comparator, odds ratio/risk ratio/hazard ratio with 95% confidence intervals, I^2^, Chi^2^ (with *p* value) and publication bias.

### Quality assessment of systematic reviews

The quality of the systematic review was classified by two independent reviewers (high 8–11, moderate 4–7, low 0–3) according to the JBI critical appraisal checklist for systematic reviews and research. Although the checklist is not intended as a quantitative tool, it allows to identify potential issues in the design of the systematic reviews that may affect the quality of the results. Any discrepancies between the reviewers were discussed, and if consensus could not be reached, a third reviewer made the final determination on study quality.

### Grading the strength and certainty of the evidence

An initial grading of the strength of the association was based on the assessment of the size of the reported effect (odds/risk ratio) according to Rosenberg and Handler [7]. The classification was as follows: none (1.0–1.2), weak (1.2–1.5), moderate (1.5–3.0), and strong (3.0–10.0).

The quality and certainty of the evidence was assessed according to the Grading of Recommendations, Assessment, Development and Evaluations (GRADE) guide. If the systematic review did not include a GRADE assessment, one was performed based on the published data.

### Synthesis of effect measurement

All study data were tabulated and presented in the qualitative synthesis. The effect measurement was the reported odds ratio (OR)/relative risk (RR)/hazard ratio (HR) with its 95% confidence interval for each NCD. The Chi² test was used to determine heterogeneity among the included studies, and the I² statistic was applied to estimate its impact. The I² value was categorized as follows: 0–40% indicating mild heterogeneity; 30–60% indicating moderate heterogeneity; 50–90% indicating substantial heterogeneity; and 75–100% indicating very considerable heterogeneity.

The measurements of effect size were plotted according to the strength of the association for each systematic review and NCD.

## Results

### General characteristics of the systematic reviews

**Fig. 1:**
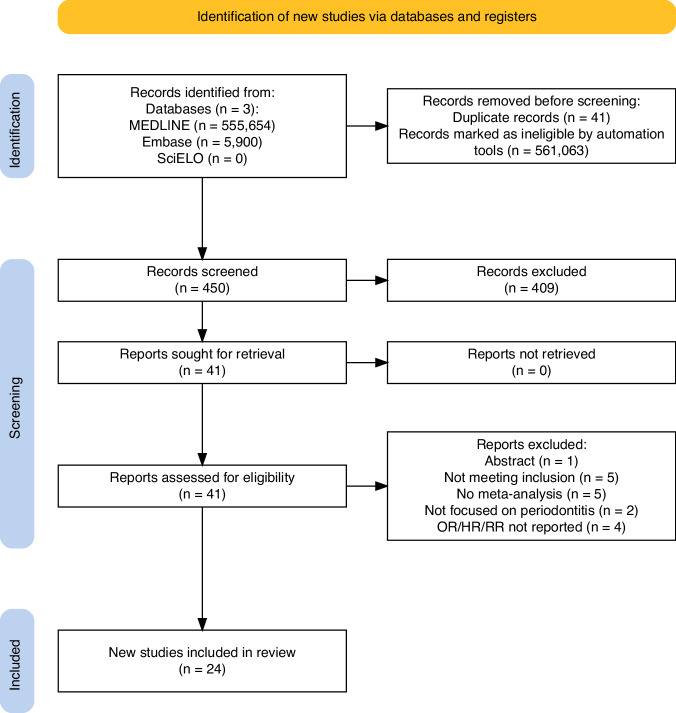
Flow diagram of systematic reviews selection.

Initially, 561,554 potential results were identified. After removing duplicates and excluding records deemed ineligible by automated filters, 450 results were screened by title and abstract. This process led to 41 records being appraised in full-text. Of these, 17 were further excluded (see supplementary material 3), leaving a total of 24 systematic reviews [8–31] that met the inclusion criteria (Fig. 1).

Table 1 lists the general characteristics of the included systematic reviews. In total, 32 NCDs were studied and consolidated: pregnancy complications (preterm birth, low birth weight, preeclampsia), cognitive impairment, cardiovascular complications (heart failure, stroke, myocardial infarction, coronary heart disease, atrial fibrillation, atrial flutter), cancers (oral, lung, colorectal, urogenital, gastric, pancreatic), gastrointestinal disease (inflammatory bowel disease, Crohn’s disease, ulcerative colitis), Sjogren’s syndrome, sleep disordered breathing, obesity, dyslipidemia, diabetes, hyperglycaemia, liver disease, osteoporosis, rheumatoid arthritis, chronic obstructive pulmonary disease, metabolic syndrome, systemic lupus erythematosus and kidney disease. In general, the majority of the systematic reviews were registered and publication lag time was 1 year on average. The methods of determining the diagnosis of the NCDs and periodontitis varied among studies but in general used clinical examination, the International Classification of Diseases codes, well established criteria, national records and self-reporting. The number of included studies in the systematic reviews ranged from 3 to 33 and publication bias was evident.

**Table 1:** General characteristics of the included systematic reviews.

Author/year	Register	Country	Disease	Definition of disease	Definition of periodontitis	Last search	Number of included studies	Number of studies high RoB	Pub bias analysis
Castaño-Suarez et al. 2024	Open Science Framework	Colombia	Preterm birth	Birth before 37 weeks	Clinical examination or periodontal indices	2023	33	4	Yes
			Low birth weight	Birth weight less than 2500 g	Clinical examination or periodontal indices				
Lin et al. 2024	PROSPERO (CRD42023395297)	China	Cognitive impairment	MMSE, MoCA, The Winblad criteria, NINCDS-ADRDA	Clinical examination or periodontal indices	2023	7	0	No
Dewan et al. 2024	PROSPERO (CRD42021237995)	India	Stroke	Ischemic stroke, hemorrhagic stroke and transient ischemic attacks	CAL > 3 mm and more than 30% of sites involved	2022	36	0	No
Ma et al. 2024	PROSPERO (CRD42023407582)	China	Oral cancer, oral squamous cell carcinoma, oropharyngeal cancer	ICD for oncology	International Classification of Diseases, Ninth Revision, Clinical Modification (ICD-9-CM)	2023	16	2	Yes
Aguiar et al. 2024	PROSPERO (CRD42021221317)	Brazil	Gastric adenocarcinoma	Self report, medical records, ICD, national registry (histology)	Self report, positive diagnosis, dental history	2022	9	0	Yes
Wang et al. 2024	PROSPERO (CRD42024502981)	China	Inflammatory bowel disease	ICD, Rare and Intractable Diseases	ICD, clinical examination, self reported	2024	6	2	Yes
			Crohn’s disease	ICD, Rare and Intractable Diseases	ICD, clinical examination, self reported				
			Ulcerative colitis	ICD, Rare and Intractable Diseases	ICD, clinical examination, self reported				
Leelaviwat et al. 2024	Not stated	USA	Heart failure	Self reported, ICD, European Society of Cardiology	Periodontal profile class, CDC criteria	2023	3	ND	No
Tan et al. 2024	PROSPERO (CRD42021272876)	Singapore	Systemic lupus erythematosus (SLE)	Systemic Lupus International Collaborating Clinics Criteria; American College of Rheumatology	Periodontal parameters and periodontal indices	2023	43	0	Yes
			Rheumatoid arthritis (RA)	American College of Rheumatology	Periodontal parameters and periodontal indices				
Verma et al. 2023	PROSPERO (CRD42023390819)	India	Lung cancer	(AECG), the European Community criteria, Copenhagen criteria	Clinical examination or periodontal indices	2022	7	0	Yes
Larvin et al. 2023	PROSPERO (CRD42019154897)	United Kingdom	Diabetes	Clearly defined classification of metabolic, autoimmune or inflammatory diseases	Clinically or self-reported diagnosis or signs of periodontitis	2022	30	17	Yes
			Kidney disease	Clearly defined classification of metabolic, autoimmune or inflammatory diseases	Clinically or self-reported diagnosis or signs of periodontitis				
			Liver disease	Clearly defined classification of metabolic, autoimmune or inflammatory diseases	Clinically or self-reported diagnosis or signs of periodontitis				
			Osteoporosis	Clearly defined classification of metabolic, autoimmune or inflammatory diseases	Clinically or self-reported diagnosis or signs of periodontitis.				
			Rheumatoid arthritis	Clearly defined classification of metabolic, autoimmune or inflammatory diseases	Clinically or self-reported diagnosis or signs of periodontitis				
Yang et al. 2023	Not stated	China	Chronic obstructive pulmonary disease (COPD)	FEV1, FVC, GOLD, self reported	Clinical examination or periodontal indices	2023	22	4	Yes
Rosário-Dos-Santos et al. 2023	PROSPERO (CRD42021232120)	Brazil	Metabolic syndrome (MetS)	NCEP ATP III, IDF, AHA/NHLBI	Partial or full oral examination, periodontal indices	2022	14	0	Yes
Leelaviwat et al. 2023	Not stated	USA	Atrial fibrillation (AF) and atrial flutter (AFL)	ICD or by a standard 12-lead ECG	ICD-9 M codes for periodontitis, Periodontal profile class	Not stated	4	ND	No
Leng et al. 2023	PROSPERO (CRD42022333663)	China	Cardiovascular disease including: myocardial infarction (MI), coronary heart disease (CHD), or stroke in MEN	ICD	Clinical or self-report	2022	26	1	Yes
			Cardiovascular disease including: myocardial infarction (MI), coronary heart disease (CHD), or stroke in WOMEN	ICD	Clinical or self-report	2022	26	1	Yes
Yang et al. 2023	PROSPERO (CRD42021261322)	China	Sjogren´s syndrome	European community criteria, The Copenhagen criteria, American-European consensus group	Periodontal parameters and periodontal indices	2021	21	5	Yes
Liu et al. 2023	Not stated	China	Sleep-disordered breathing (Obstructive sleep apnea)	PSG	Periodontal examination (AAP/CDC definitions)	2022	10	0	Yes
Le et al. 2022	Not stated	Australia	Preeclampsia	blood pressure of ≥140/90 mmHg after 20 weeks of gestation, combined with proteinuria of at least 1+on midstream urine specimen or catheter specimen	≥ 2 sites with PD ≥ 4 mm and CAL ≥ 3 mm, not on the same site or one site with PD ≥ 5 mm at the same site or evaluated the progression by clinical periodontal parameters	Not stated	30	9	Yes
Xu et al. 2022	Not stated	China	Pancreatic cancer	ICD	ICD, clinical examination, bone loss	2021	17	6	Yes
Li et al. 2022	PROSPERO (CRD42021244405)	China	Urogenital cancer	Medical records, pathology, ICD, cancer registry	Questionnaires, clinical periodontal examinations and radiographic examinations or other credible forms of medical records were acceptable	2022	11	0	Yes
Mirzaei et al. 2022	Not stated	Iran	Dyslipidemia	TG > 200 mg/dl, TC > 200 mg/dl, HDL < 60 mg/dl, LDL > 30 mg/dl, HDL < 40 mg/dl (men), HDL < 50 mg/dl (women), TG ≥ 150 mg/dl	CAL > 1, PD > 2, and CPI > 2	2021	31	2	Yes
Kim et al. 2022	PROSPERO (CRD42022301343)	South Korea	Obesity	BMI ≥ 30 kg/m2 or higher, but BMI ≥ 25 kg/m^2^ in Asians. Waist circumference as ≥88 cm in women and ≥102 cm in men, but ≥90 cm for men and ≥80 cm for women in Asians	(PPD) ≥ 4 mm, clinical attachment level (CAL) ≥ 1 mm, and community periodontal index (CPI) ≥ 3	2022	37	2	Yes
Li et al. 2021	Not stated	China	Colorectal cancer	ICD	Self reported, CDC-AAP, Russell index, radiographs	2020	7	2	Yes
Mirzaei et al. 2021	Not stated	Iran	Hyperglycaemia	HbA1c > 6% or 7.5% or 9%; Fasting blood glucose >100 or 110 or 120 or 126 mg/dL	Periodontal parameters and periodontal indices	2021	19	0	Yes
Zheng et al. 2021	PROSPERO (CRD42019128053)	China	Type 2 diabetes mellitus	HbA1c > 6.7% or 7%; Fasting blood glucose >126 mg/dL	Periodontal parameters	2020	40	2	Yes

*RoB* risk of bias, *MMSE* Mini-Mental State Examination, *MoCA* Montreal Cognitive Assessment, *The Winblad criteria, NINCDS-ADRDA* National Institute of Neurological and Communicative Disorders and Stroke and the Alzheimer’s Disease and Related Disorders Association, *ICD* International Classification of Diseases, *AECG* American-European Consensus Group Criteria, *FEV1* forced expiratory volume in 1s, *FVC* forced vital capacity, *GOLD* Global Initiative for Chronic Obstructive Lung Disease, *NCEP ATP III* National Cholesterol Education Program - Adult Treatment Panel III, *IDF* International Diabetes Federation, *AHA/NHLBI* American Heart Association and the National Heart Lung and Blood Institute, *ECG* Electrocardiogram, *PSG* polysomnography, *TG* triglycerides, *TC* total cholesterol, *HDL* high density lipoprotein, *LDL* low density lipoprotein, *BMI* body mass index, *HbA1C* glycated hemoglobin, *PD* probing depth, *CAL* clinical attachment loss, *AAP* American academy of periodontology, *CDC* Centers for disease control.

**Fig. 2:**
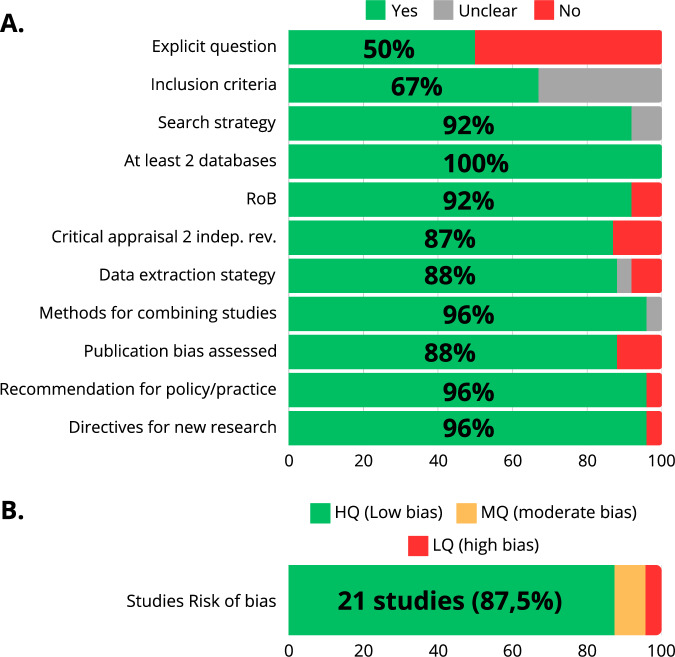
Risk of bias assessment of included systematic reviews. **A** Shows the frequency distribution of the quality domains. **B** Shows the general risk of bias of the systematic reviews.

The risk of bias assessment indicated that 21 systematic reviews (87.5%) demonstrated low bias (high quality) [8–13, 15–19, 21–27, 29–31], 2 had medium bias [14, 28], and 1 exhibited high bias (low quality) [20]. Key issues identified included the formulation of explicit research questions, critical appraisal, data extraction, and publication bias (Fig. 2 and Supplementary Material 4). Two systematic reviews with meta analysis were published in the format of rapid communication and therefore lacked some information in the methods [14, 20]. However, they were kept since they analyzed the influence of periodontitis on two cardiovascular conditions that were not identified in other reviews.

### The association between periodontitis and NCDs

**Fig. 3:**
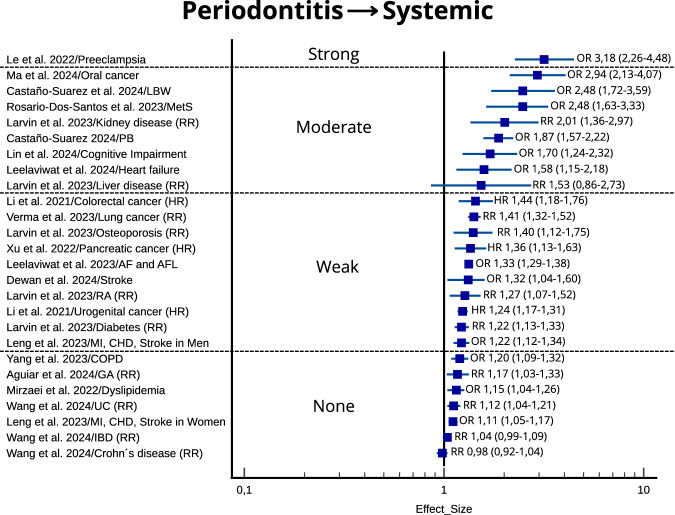
Plot of the strength of the association according to the size of the effect for periodontitis → noncommunicable diseases. OR odds ratio, HR hazard ratio. 95% confidence interval is shown in parenthesis. LBW low birth weight, MetS metabolic syndrome, PB preterm birth, AF atrial fibrillation, AFL atrial flutter, RA rheumatoid arthritis, MI myocardial infarction, CHD coronary heart disease, COPD chronic obstructive pulmonary disease, GA gastric adenocarcinoma, UC ulcerative colitis, IBD inflammatory bowel disease.

The strength of the association between periodontitis and NCDs is presented in Fig. 3. The estimate of the association was strong in 1 systematic review (preeclampsia), moderate in 8 (oral cancer, low birth weight, metabolic syndrome, kidney disease, preterm birth, cognitive impairment, heart failure, liver disease), weak in 10 (colorectal cancer, lung cancer, osteoporosis, pancreatic cancer, atrial fibrillation and flutter, stroke, rheumatoid arthritis, urogenital cancer, diabetes, cardiovascular complications in men), and absent in 7 systematic reviews (chronic obstructive pulmonary disease, gastric adenocarcinoma, dyslipidemia, ulcerative colitis, cardiovascular complications in women, inflammatory bowel disease, Crohn’s disease). The size of the reported effect was broader with increasing strength and this should be interpreted cautiously. Periodontitis significantly increases the odds of preeclampsia (OR 3.18; 95% CI 2.26–4.48), with the confidence interval not including 1, though it appears somewhat wide. This suggests that there is substantial evidence supporting an association between periodontitis and preeclampsia. Conversely, the relative risk (RR) of liver disease is 1.53 (95% CI 0.86–2.73), with a confidence interval that includes 1 and is wide, indicating a moderate but unstable association between periodontitis and liver disease. Finally, the odds ratio for stroke, myocardial infarction, and coronary heart disease in women is 1.11 (95% CI 1.05–1.17), which does not support an association between periodontitis and these cardiovascular events.

### The association between NCDs and periodontitis

**Fig. 4:**
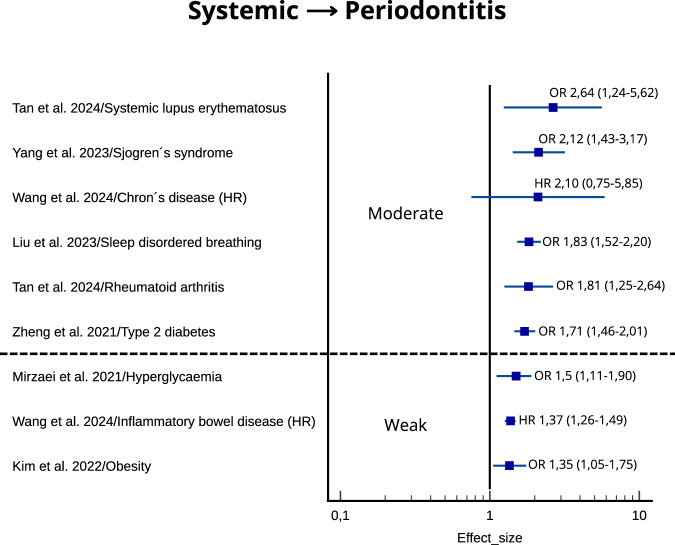
Plot of the strength of the association according to the size of the effect for noncommunicable diseases → periodontitis. OR odds ratio, HR hazard ratio. 95% confidence interval is shown in parenthesis.

The strength of the association between NCDs and periodontitis is depicted in Fig. 4. The estimate of the association was moderate in 6 systematic reviews (systemic lupus erythematosus, Sjogren’s syndrome, Crohn’s disease, sleep disordered breathing, rheumatoid arthritis, type 2 diabetes) and weak in 3 systematic reviews (hyperglycaemia, inflammatory bowel disease, obesity). In general, the reported size of the effect was broader also with increasing strength, which as result impacted the precision of the certainty of the evidence. In summary, the evidence suggests that certain NCDs are associated with periodontitis, but the wider confidence intervals indicate that these findings should be interpreted with caution.

### Certainty of the evidence for the bidirectional association between periodontitis and NCDs

Although data supports the association between periodontitis and NCDs, and to a lesser extent between some NCDs and periodontitis, the certainty of the evidence was classified as low to very low (Tables 2 and 3). This means that our confidence in the effect estimates is very limited and the actual effect is likely to be substantially different from the effect estimate. It is important to note that the level of certainty was primarily downgraded due to potential risks of bias, as well as heterogeneity in both the effect sizes and study methodologies, which impacted the precision of the results.

**Table 2:** GRADE assessment of the evidence for periodontitis → noncommunicable diseases.

Year/author	Disease	Design	Risk of bias	Inconsistency	Indirectness	Imprecision	Pub Bias	Other considerations	Certainty	Importance
Aguiar et al. 2024	Gastric adenocarcinoma	9 analytical studies	Serious. Several studies carried a large effect	Not serious. Heterogeneity 41.5%	Serious. Information regarding representativeness and comparability of groups was detected	Serious. Confidence intervals are wide in some studies	Not serious	7/9 studies showed no association between periodontitis and gastric adenocarcinoma	Very low	Important
Castaño-Suarez 2024	Preterm birth	25 analytical studies	Serious. 4/25 studies carried great weight to the effect. 11/25 studies considered as moderate/high bias	Serious: Heterogeneity 95%, Chi2 510.1	Serious. Information regarding selection of participants is lacking in some studies	Serious. Confidence intervals are wide in some studies	Serious. Publication bias detected	Some of the studies demonstrated a higher prevalence of periodontitis in the case group compared to the control group	Low	Important
Castaño-Suarez 2024	Low birth weight	20 analytical studies	Serious. 4/20 studies carried great weight to the effect. 4/20 studies considered as moderate/high bias	Serious: Heterogeneity 93%, Chi2 279.9	Serious. Information regarding selection of participants is lacking in some studies	Serious. Confidence intervals are wide in some studies	Serious. Publication bias detected	Some of the studies demonstrated a higher prevalence of periodontitis in the case group compared to the control group	Low	Important
Dewan et al. 2024	Stroke	13 analytical studies	7/13 studies carried a large effect. risk of bias low to moderate	Not serious. Heterogeneity 30.3%	Serious. Information regarding representativeness and comparability of groups was detected	Serious. Confidence intervals are wide in some studies	Not performed	2/13 studies showed no association between periodontitis and stroke	Low	Important
Leelaviwat et al. 2024	Heart failure	3 cohort studies	Serious. risk of bias not reported	Serious. Heterogeneity 78.4%	Incomplete information.	Serious. Confidence intervals are wide in some studies	Not performed	Incomplete information. No RoB analyses and GRADE	Very low	Important
Lin et al. 2024	Cognitive impairment	7 analytical studies	Serious. 3/7 studies carried great weight to the effect. 5/7 studies considered as moderate bias	Not serious. heterogeneity 0%. Chi2 5.79	Not serious	Serious. Confidence intervals are wide in some studies	Publication bias analysis not performed due to low number of studies	5/7 studies showed no association between periodontitis and cognitive impairment	Very low	Important
Ma et al. 2024	Oral cancer, oral squamous cell carcinoma, oropharyngeal cancer	9 case control studies	Serious. 2 studies high risk of bias	Not serious. Heterogeneity 52.4%.	Serious. Information regarding representativeness and comparability of groups was detected	Serious. Confidence intervals are wide in some studies	Not serious	2/9 studies showed no association between periodontitis and oral cancer	Low	Important
Wang et al. 2024	Inflammatory bowel disease	4 cohort studies	Serious. Risk of bias detected in all studies	Not serious. Heterogeneity 27%	Serious. Information regarding representativeness and comparability of groups was detected	Serious. Confidence intervals are wide in some studies	Not serious	Studies show no association between periodontitis and IBD	Very low	Important
Wang et al. 2024	Crohn’s disease	4 cohort studies	Serious. Risk of bias detected in all studies	Not serious. Heterogeneity 0%	Serious. Information regarding representativeness and comparability of groups was detected	Serious. Confidence intervals are wide in some studies	Not serious	Studies show no association between periodontitis and CD	Very low	Important
Wang et al. 2024	Ulcerative colitis	4 cohort studies	Serious. Risk of bias detected in all studies	Not serious. Heterogeneity 38%	Serious. Information regarding representativeness and comparability of groups was detected	Serious. Confidence intervals are wide in some studies	Not serious	Some studies showed no association between periodontitis and UC. Otherwise the association is very close to 1	Very low	Important
Larvin et al. 2023	Diabetes	9 analytical studies	Serious	Not serious. heterogeneity 53.8%. Q 20.21	Serious. Information regarding selection of participants is lacking in some studies	Serious. Confidence intervals are wide in some studies	Publication bias suspected	3/9 studies showed no association between periodontitis and incident diabetes. Otherwise the association is close to 1	Low	Important
Larvin et al. 2023	Kidney disease	2 analytical studies	Very serious	Not serious. heterogeneity 0%	Serious. Information regarding selection of participants is lacking in some studies	Serious. Confidence intervals are wide in some studies	Publication bias suspected	Both studies showed significant association between periodontitis and kidney disease	Very low	Important
Larvin et al. 2023	Liver disease	3 analytical studies	Very serious. 1/3 studies carried a large effect. Confounding factors are important.	Serious: Heterogeneity 91.6%, Q 13.55	Serious. Information regarding selection of participants is lacking in some studies	Serious. Confidence intervals are wide in some studies	Publication bias suspected	The overall shows no association between periodontitis and liver disease	Very low	Important
Larvin et al. 2023	Osteoporosis	4 analytical studies	Very serious. 2/4 carried a large effect. Confounding factors are important.	Serious: Heterogeneity 93%, Q 45.07	Serious. Information regarding selection of participants is lacking in some studies	Serious. Confidence intervals are wide in some studies	None	All studies show association between periodontitis and osteoporosis	Very low	Important
Larvin et al. 2023	Rheumatoid arthritis	4 analytical studies	Very serious. Confounding factors are important.	Serious. Heterogeneity 72.8%, Q 12.66	Serious. Information regarding selection of participants is lacking in some studies	Not serious	None	All studies show association between periodontitis and RA	Low	Important
Leelaviwat et al. 2023	Atrial fibrillation (AF) and atrial flutter (AFL)	3 cohort studies	Unknown since it was not mentioned in the article	Not serious. heterogeneity 3%	Unknown since it was not mentioned in the article	Not serious	Unknown since it was not mentioned in the article	Unknown since it the methods section is very short. Although the confidence interval is narrow, the value is close to 1	Very low	Important
Leng et al. 2023	Myocardial infarction (MI), coronary heart disease (CHD), or stroke in MEN	17 analytical studies in men	Not serious. 1 study high risk of bias	Serious. Heterogeneity 93%, Chi2 243.4	Serious. Information regarding representativeness and comparability of groups was detected	Serious. Confidence intervals are wide in some studies	Not serious	8/17 studies showed no association between periodontitis and CVD. Otherwise the association is close to 1	Very low	Important
Leng et al. 2023	Myocardial infarction (MI), coronary heart disease (CHD), or stroke in WOMEN	9 analytical studies in women	Not serious. 1 study high risk of bias	Serious. Heterogeneity 85%, Chi2 95.8	Serious. Information regarding representativeness and comparability of groups was detected	Serious. Confidence intervals are wide in some studies	Not serious	Some studies showed no association between periodontitis and CVD. Otherwise the association is very close to 1	Very low	Important
Rosário-Dos-Santos et al. 2023	Metabolic syndrome (MetS)	6 analytical studies moderate-severe periodontitis	Serious. Sources of bias included lack of clarity in non-respondents, missing data and sample size calculation. 1/6 studies carried a large effect.	Serious.Heterogeneity 86.1%	Serious: information regarding representativeness of sample population and comparability is lacking	Not serious	Publication bias suspected	1/6 studies showed no association between periodontitis and MetS	Low	Important
Verma et al. 2023	Lung cancer	7 analytical studies	1/7 studies carried a large effect. 3/7 studies considered medium risk of bias	Serious: Heterogeneity 82%, Chi2 33.9	Serious. Information regarding selection of participants is lacking in some studies	Serious. Confidence intervals are wide in some studies	Publication bias performed with less than 10 studies	2/7 studies showed no association between periodontitis and lung cancer. Otherwise the association is close to 1	Very low	Important
Yang et al. 2023	Chronic obstructive pulmonary disease (COPD)	22 analytical studies	Serious. 3/22 studies carried a large effect and risk of bias high in 4 studies	Serious: Heterogeneity 79%, Chi2 86.21	Serious. Information regarding representativeness of sample population and comparability is lacking	Serious. Confidence intervals are wide in some studies	None	9/22 studies showed no association between periodontitis and COPD	Low	Important
Le et al. 2022	Preeclampsia	28 analytical studies	Very serious. 8/28 studies carried large effect	Serious: Heterogeneity 81%, Chi2 139.03	Serious. Information regarding comparability is lacking in some studies	Serious. Confidence intervals are wide in some studies	None	9/28 studies showed no association between periodontitis and preeclampsia	Low	Important
Li et al. 2022	Urogenital cancer	11 analytical studies	Serious. Several studies carried a large effect.	Not serious. Heterogeneity 22.4%	Serious. Information regarding representativeness and comparability of groups was detected.	Serious. Confidence intervals are wide in some studies	Not serious	5/11 studies showed no association between periodontitis and urogenital cancer. Otherwise the association is close to 1	Very low	Important
Mirzaei et al. 2022	Dyslipidemia	13 analytical studies	Serious. Moderate risk of bias detected in most studies	Not serious. Heterogeneity 28.7%	Serious. Information regarding representativeness and comparability of groups was detected.	Not serious	Not serious		Very low	Important
Xu et al. 2022	Pancreatic cancer	14 analytical studies	Serious. High risk of bias in 6 studies	Serious. Heterogeneity 60.8%	Serious. Information regarding representativeness and comparability of groups was detected	Serious. Confidence intervals are wide in some studies	Publication bias suspected	Not serious	Very low	Important
Li et al. 2021	Colorectal cancer	7 cohort studies	Serious. Several studies carried a large effect.	Serious. Heterogeneity 53.9%	Serious. Information regarding representativeness and comparability of groups was detected	Serious. Confidence intervals are wide in some studies	Not serious	4/7 studies showed no association between periodontitis and colorectal cancer	Very low	Important

The Grading of Recommendations Assessment, Development and Evaluation (GRADE).

**Table 3:** GRADE assessment of the evidence for noncommunicable diseases → periodontitis.

		GRADE domains								
Year/author	Disease	Design	Risk of bias	Inconsistency (I2)	Indirectness	Imprecision	Pub Bias	Other considerations	Certainty	Importance
Tan et al. 2024	Systemic lupus erythematosus (SLE)	10 analytical studies	Serious. Some studies carried a very large effect	Serious. Heterogeneity 89%, Chi^2^ 83.6	Serious. There were issues regarding comparability and selection of participants	Serious. Confidence intervals are wide in some studies	Serious. Publication bias detected	6/10 studies showed no association between SLE and periodontitis	Very low	Important
Tan et al. 2024	Rheumatoid arthritis (RA)	29 analytical studies	Serious. 9 studies carried a very large effect	Serious. Heterogeneity 90%, Chi^2^ 282.3	Serious. There were issues regarding comparability and selection of participants	Serious. Confidence intervals are wide in some studies	Serious. Publication bias detected	13/29 studies showed no association between RA and periodontitis	Very low	Important
Wang et al. 2024	Inflammatory bowel disease	2 longitudinal studies	Serious. 1 study carried a large effect. Risk of bias was detected	Not serious. Heterogeneity 18%	Serious. There were issues regarding comparability and selection of participants	No serious. Confidence interval was wide in 1 study. However, the range of the CI was small for over 33,000 individuals	Not determined due to low number of studies	Heterogeneity in study methods may act as a confounding factor	Very low	Important
Wang et al. 2024	Crohn’s disease	2 longitudinal studies	Serious. 1 study carried a large effect. Risk of bias was detected	Serious. Heterogeneity 81%	Serious. There were issues regarding comparability and selection of participants	Serious. Confidence interval was wide in 1 study	Not determined due to low number of studies	Possible false negatives for CD according to the RIS threshold (required information size)	Very low	Important
Yang et al. 2023	Sjogrens	5 analytical studies	Serious. 1/5 studies carried a very large effect	Serious. Heterogeneity 77.5%	Serious. There were issues regarding comparability and selection of participants	Serious. Confidence interval was large in one study	Not serious	3/5 studies showed association between Sjogrens syndrome and periodontitis	Low	Important
Liu et al. 2023	Sleep-disordered breathing (Obstructive sleep apnea)	10 analytical studies	Serious. 4 studies carried a large effect	Not serious. heterogeneity 40%, Chi^2^ 16.6	Serious. There were issues regarding comparability and selection of participants	Serious. Confidence intervals wide in 4 studies	Not serious	4/10 studies showed no association between SDB and periodontitis. Heterogeneity in study methods may act as a confounding factor	Very low	Important
Kim et al. 2022	Obesity	29 analytical studies	Serious. Several studies carried a large effect. Risk of bias was moderate to low	Serious. Heterogeneity 98%, Chi^2^ 1356.12	Serious. There were issues regarding comparability and selection of participants	Serious. Confidence interval are wide in several studies	Not serious	12/29 studies showed no association between obesity and periodontitis	Very low	Important
Mirzaei et al. 2021	Hyperglycaemia	11 cross-sectional studies	Not serious	Serious. Heterogeneity 84.1%	Not serious	Not serious	Serious. Publication bias detected	Most studies showed association between hyperglycemia and periodontitis	Low	Important
Zheng et al. 2021	Type 2 diabetes mellitus	16 analytical studies	Serious. 5/16 studies carried a large effect. Studies rated high and moderate risk of bias	Not serious. Heterogeneity 32%, Chi^2^ 22.05	Serious. There were issues regarding comparability and selection of participants	Serious. Confidence intervals are wide in some studies although combined CI was small	Not serious	10/16 studies showed no association between diabetes and periodontitis. Heterogeneity in study methods may act as a confounding factor	Low	Important

High quality: We are very confident that the real effect corresponds to the effect estimate. Moderate quality: We are moderately confident in the effect estimate; The true effect is likely to be close to the effect estimate, but there is a possibility that it will be substantially different. Low quality: our confidence in the effect is limited; the actual effect may be substantially different from the estimated effect. Very low quality: we have very little confidence in the effect estimate; The actual effect is likely to be substantially different from the effect estimate.

The Grading of Recommendations Assessment, Development and Evaluation (GRADE).

## Discussion

The results of this umbrella review objectively compile the best available evidence regarding the bidirectional association between periodontitis and systemic health. However, even the highest quality evidence may not always be sufficient to draw definitive conclusions that inform clinical decision-making. To date, aside from the review by Botelho et al. [32], this study is the most recent and specifically addresses the grading of evidence certainty in this area. In addition, it does not focus on biological mechanisms but rather examines data derived from various analytical studies in humans.

### Significance of the results

Is periodontitis a risk factor for systemic health complications and vice versa, or is it merely coincidental? The results of this umbrella review indicate that while periodontitis is associated with certain NCDs, it does not establish a causal relationship. The most plausible explanation is that periodontitis and systemic health issues often occur simultaneously and share common inflammatory and microbiological mechanisms. Consequently, the presence of one condition may influence the development of the other. The studies included in this review highlighted the co-occurrence of multiple systemic conditions and confounding factors. Therefore, it is not possible to confirm that periodontitis independently contributes to systemic health complications and vice versa.

Multimorbidity is the presence of two or more chronic conditions in an individual, with no one ailment being regarded as the dominant focus. This phenomenon is becoming more common in aging populations and poses considerable challenges to healthcare systems because of the complexities of addressing many, often interconnected, diseases at the same time. Multimorbidity complicates clinical decision-making while also increasing the strain on patients, who must navigate many therapies and healthcare providers. It is linked to higher healthcare expenses, lower quality of life, and increased mortality, emphasizing the importance of integrated and patient-centered approaches to care. Large population-based studies have demonstrated that a significant proportion of adults—27%—report having two or more chronic conditions, with the prevalence increasing notably with advancing age [33, 34]. In our umbrella review, periodontitis appears to be associated with some systemic chronic diseases (i.e. systemic lupus erythematosus to periodontitis or periodontitis to preeclampsia) and tend to co-occur, primarily because they share common inflammatory mechanisms, as well as genetic, environmental, and sociodemographic determinants. Therefore, periodontitis remains a significant condition in the context of multimorbidity because it has the ability to contribute to and be affected by several chronic diseases. Understanding the link between periodontitis and multimorbidity is critical for establishing holistic care methods that target the underlying causes of these non-communicable chronic diseases to reduce the health impact of aging.

### Limitations of this review and perspective

The main limitation of this umbrella review is that the quality of the evidence is inherently influenced by the quality of the original research. Inconsistencies and contradictions among the included studies can complicate the interpretation of the overall findings. Each systematic review identified potential sources of bias, which must be considered when evaluating the results. Consequently, the certainty of the evidence was rated as low. This suggests that the conclusions drawn from the studies should be approached with caution, given the challenges associated with investigating the interactions between periodontitis and systemic conditions in humans.

To enhance the quality of data supporting the link between periodontitis and NCDs, future research should focus on well-designed longitudinal studies that minimize bias and appropriately consider multimorbidity. This involves utilizing statistical models that account for the effects of multiple chronic diseases, as well as employing standardized diagnostic tools. Additionally, to address health disparities and improve overall population health, it is essential to understand how socioeconomic, racial, and ethnic factors influence the prevalence, severity, and accessibility of treatment for both systemic diseases and periodontitis.

## Conclusions

There is some data that, with varying degrees of association and low to very low certainty, provide evidence that periodontitis may be a potential risk factor for some NCDs and vice versa.

## Supplementary information

Supplementary material 1-3

Supplementary material 4

Prisma checklist
