# Supplementary material for: Grading the strength and certainty of the scientific evidence of the bidirectional association between periodontitis and noncommunicable diseases: an umbrella review
Source: Evid Based Dent. 2025 Mar 13;26(3):147. doi: 10.1038/s41432-025-01132-9 (PMC12474539; doi:10.1038/s41432-025-01132-9)
Supplement: Supplementary file 1 — Supplementary material 1-3 [file 41432_2025_1132_MOESM1_ESM.docx]

Supplementary material 1. List of noncommunicable diseases that have been associated with periodontitis according to artificial intelligence (AI- ChatGPT 4.0)

| **Prompt:** ¨List all the systemic conditions that have been associated with periodontitis according to the scientific evidence. If possible, in order of importance.¨ |
| --- |
| Periodontitis, a severe gum infection that damages the soft tissue and bone supporting the teeth, has been associated with various systemic conditions. The association is believed to be bidirectional, where systemic conditions may exacerbate periodontitis and vice versa. Here is a list of systemic conditions associated with periodontitis, generally listed in order of the strength and consistency of scientific evidence:  1. **Cardiovascular Diseases**:  - Coronary artery disease  - Atherosclerosis  - Stroke  2. **Diabetes Mellitus**:  - Type 1 Diabetes  - Type 2 Diabetes  - Hyperglycemia  3. **Adverse Pregnancy Outcomes**:  - Preterm birth  - Low birth weight  - Preeclampsia  4. **Respiratory Diseases**:  - Chronic obstructive pulmonary disease (COPD)  - Pneumonia severe covid19 symptoms  5. **Rheumatoid Arthritis**:  - Increased severity of rheumatoid arthritis symptoms  6. **Chronic Kidney Disease**:  - Worse progression of kidney disease  7. **Obesity**:  - Increased risk of periodontal disease  8. **Cognitive Disorders**:  - Alzheimer's disease  - Dementia  9. **Metabolic Syndrome**:  - Cluster of conditions including high blood pressure, high blood sugar, excess body fat around the waist, and abnormal cholesterol levels  10. **Osteoporosis**:  - Reduced bone density and increased risk of fractures  11. **Cancer**:  - Higher risk of certain cancers, such as pancreatic cancer  12. **Inflammatory Bowel Disease**:  - Crohn’s disease  - Ulcerative colitis  13. **Psoriasis**:  - Chronic skin condition  14. **Non-alcoholic Fatty Liver Disease (NAFLD)**:  - Liver inflammation and damage  15. **Endocarditis**:  - Infection of the inner lining of the heart  16. **HIV/AIDS**:  - Compromised immune system leading to increased susceptibility to periodontal disease  17. **Anemia**:  - Iron-deficiency anemia and other types impacting oral health  18. **Sjogren´s syndrome  The strength of the association varies, and while some conditions like cardiovascular disease and diabetes have well-established links with periodontitis, others require more research for definitive conclusions. |

Supplementary material 2. Keywords used for the systematic search.

| **Keyword for periodontitis** | **Connector** | **Keyword for systemic outcomes** |
| --- | --- | --- |
| Periodontitis | AND | Cardiovascular Diseases OR Coronary artery disease OR Atherosclerosis OR diabetes OR adverse pregnancy outcomes OR Preterm birth OR Low birth weight OR Preeclampsia OR Chronic obstructive pulmonary disease (COPD) OR Pneumonia OR rheumatoid arthritis OR kidney disease OR obesity OR metabolic syndrome OR Cognitive Disorders OR Alzheimer OR Dementia OR osteoporosis OR cancer OR endocarditis OR  Inflammatory Bowel Disease OR Crohn’s disease OR Ulcerative colitis OR Sjogren OR Psoriasis OR Non-alcoholic Fatty Liver Disease (NAFLD) OR Liver inflammation OR anemia |

Supplementary material 3. List of excluded studies with reasons.

| **Reference** | **Reason** |
| --- | --- |
| Triebl Z, Bencze B, Bányai D, Rózsa N, Hermann P, Végh D. Poor glycemic control impairs oral health in children with type 1 diabetes mellitus - a systematic review and meta-analysis. BMC Oral Health. 2024 Jun 28;24(1):748. doi: 10.1186/s12903-024-04516-y. PMID: 38943074; PMCID: PMC11212250. | Not meeting inclusion |
| Trzcionka A, Mączkowiak D, Korkosz R, Rahnama M, Duława J, Tanasiewicz M. Oral Findings in Hemodialyzed Patients Diagnosed with Diabetes Mellitus and/or Hypertension-A Systematic Review. J Clin Med. 2023 Nov 13;12(22):7072. doi: 10.3390/jcm12227072. PMID: 38002685; PMCID: PMC10671938. | No metaanalysis |
| Zhao D, Sun Y, Li X, Wang X, Lu L, Li C, Pan Y, Wang S. Association between Periodontitis and HbA1c Levels in Non-Diabetic Patients: A Systematic Review and Meta-Analysis. Healthcare (Basel). 2023 Sep 28;11(19):2649. doi: 10.3390/healthcare11192649. PMID: 37830686; PMCID: PMC10572398. | Not meeting inclusion |
| Alwithanani N. Periodontal Diseases and Diabetes Mellitus: A Systematic Review. J Pharm Bioallied Sci. 2023 Jul;15(Suppl 1):S54-S63. doi: 10.4103/jpbs.jpbs_515_22. Epub 2023 Jul 5. PMID: 37654263; PMCID: PMC10466651. | Incomplete data metaanalysis |
| Mohseni Homagarani Y, Adlparvar K, Teimuri S, Tarrahi MJ, Nilchian F. The effect of diabetes mellitus on oral health-related quality of life: A systematic review and meta-analysis study. Front Public Health. 2023 Feb 24;11:1112008. doi: 10.3389/fpubh.2023.1112008. PMID: 36908413; PMCID: PMC9998896. | Not focused on periodontitis |
| Tsikouras P, Oikonomou E, Nikolettos K, Andreou S, Kyriakou D, Damaskos C, Garmpis N, Monastiridou V, Nalmpanti T, Bothou A, Iatrakis G, Nikolettos N. The Impact of Periodontal Disease on Preterm Birth and Preeclampsia. J Pers Med. 2024 Mar 26;14(4):345. doi: 10.3390/jpm14040345. PMID: 38672972; PMCID: PMC11051368. | No metaanalysis |
| Roberts M, Jimson S, Srinivasan M. Are Adults Over 18 Years of Age with Anaemia More Likely to Develop Chronic Periodontitis Than Adults Without Anaemia? - A Systematic Review and Meta-Analysis. J Int Soc Prev Community Dent. 2023 Aug 30;13(4):287-298. doi: 10.4103/jispcd.JISPCD_37_23. PMID: 37876584; PMCID: PMC10593371. | Odds ratio not reported |
| da Silva BB, Pontes AEF, Lemos CAA, Ortega RM. Association between sickle cell disease and periodontal disease: A systematic review. J Periodontal Res. 2023 Aug;58(4):679-686. doi: 10.1111/jre.13129. Epub 2023 May 26. PMID: 37237445. | Not focused on periodontitis |
| Lu L, Zhao D, Li C, Sun Y, Geng F, Zhang S, Li W, Wang S, Pan Y. The role of periodontitis in the development of atherosclerotic cardiovascular disease in participants with the components of metabolic syndrome: a systematic review and meta-analysis. Clin Oral Investig. 2024 May 27;28(6):339. doi: 10.1007/s00784-024-05731-1. PMID: 38801482. | Not meeting inclusion |
| Al-Maweri SA, Alhajj MN, Halboub E, Tamimi F, Salleh NM, Al-Ak'hali MS, Kassim S, Abdulrab S, Anweigi L, Mohammed MMA. The impact of periodontal disease on the clinical outcomes of COVID-19: A systematic review and meta-analysis. BMC Oral Health. 2023 Sep 9;23(1):658. doi: 10.1186/s12903-023-03378-0. PMID: 37689665; PMCID: PMC10493030. | Not meeting inclusion |
| Rak D, Kulloli AM, Shetty SK, Tripathy S, Mathur A, Mehta V, Cicciù M, Minervini G. Correlation between rheumatoid arthritis and chronic periodontitis: a systematic review and meta-analysis. Minerva Dent Oral Sci. 2024 Jun 13. doi: 10.23736/S2724-6329.23.04891-X. Epub ahead of print. PMID: 38869834. | Odds ratio not reported |
| Gheorghe DN, Popescu DM, Dinescu SC, Silaghi M, Surlin P, Ciurea PL. Association between Sjögren's Syndrome and Periodontitis: Epidemiological, Fundamental and Clinical Data: A Systematic Review. Diagnostics (Basel). 2023 Apr 12;13(8):1401. doi: 10.3390/diagnostics13081401. PMID: 37189501; PMCID: PMC10137501. | No metaanalysis |
| Dibello V, Lobbezoo F, Solfrizzi V, Custodero C, Lozupone M, Pilotto A, Dibello A, Santarcangelo F, Grandini S, Daniele A, Lafornara D, Manfredini D, Panza F. Oral health indicators and bone mineral density disorders in older age: A systematic review. Ageing Res Rev. 2024 Jul 9;100:102412. doi: 10.1016/j.arr.2024.102412. Epub ahead of print. PMID: 38992442. | No metaanalysis |
| Qi J, Chen J, Pang Y, Guo Y, Chen G, Liu Y, Wang J, Liu E. Association between periodontal disease and osteoporosis in postmenopausal women: A systematic review and meta-analysis. Heliyon. 2023 Oct 20;9(11):e20922. doi: 10.1016/j.heliyon.2023.e20922. PMID: 37920517; PMCID: PMC10618781. | Odds ratio not reported |
| Kandaswamy E, Lee CT, Gururaj SB, Shivanaikar S, Joshi VM. Association of adipokine levels with obesity in periodontal health and disease: A systematic review with meta-analysis and meta-regression. J Periodontal Res. 2024 Aug;59(4):623-635. doi: 10.1111/jre.13263. Epub 2024 Apr 9. PMID: 38594806. | Odds ratio not reported |
| Natchaya Polpichai, Sakditad Saowapa, Panisara Fangsaard, Phuuwadith Wattanachayakul, Chalothorn Wannaphut and Manasawee Tanariyakul. Mo1191 ASSOCIATION BETWEEN PERIODONTAL DISEASE AND GASTROINTESTINAL TRACT CANCERS: A SYSTEMATIC REVIEW AND META-ANALYSIS. Gastroenterology 2024 166:5 (S-972) Supplement | Abstract |
| Said-Sadier N, Sayegh B, Farah R, Abbas LA, Dweik R, Tang N, Ojcius DM. Association between Periodontal Disease and Cognitive Impairment in Adults. Int J Environ Res Public Health. 2023 Mar 7;20(6):4707. doi: 10.3390/ijerph20064707. PMID: 36981618; PMCID: PMC10049038. | No metaanalysis |
