# Supplementary material for: Grading the strength and certainty of the scientific evidence of the bidirectional association between periodontitis and noncommunicable diseases: an umbrella review
Source: Evid Based Dent. 2025 Mar 13;26(3):147. doi: 10.1038/s41432-025-01132-9 (PMC12474539; doi:10.1038/s41432-025-01132-9)
Supplement: Supplementary file 2 — Supplementary material 4 [file 41432_2025_1132_MOESM2_ESM.pdf]

Supplementary material 4. JBI critical appraisal checklist for systematic reviews

Systematic review	Is the review question clearly and explicitly stated? P(I/E)CO question	Were the inclusion criteria appropriate for the review question?	Was the search strategy appropriate? A systematic review should provide evidence of the search strategy that has been used to locate the evidence. keywords, language, limits, years, etc.	Were the sources and resources used to search for studies adequate? At least 2 databases, grey literature, reference list, contacts	Were the criteria for appraising studies appropriate? Risk of bias o any tool to assess the quality	Was critical appraisal conducted by two or more reviewers independently	Were there methods to minimize errors in data extraction? Strategies to minimize bias may include conducting all data extraction in duplicate and independently, using specific tools or instruments to guide data extraction and some evidence of piloting or training around their use	Were the methods used to combine studies appropriate?	Was the likelihood of publication bias assessed? Explicit statement of publication bias assessed by funnel plot or eggers test	Were recommendations for policy and/or practice supported by the reported data? In the discussion: Is there evidence that the strength of the findings and the quality of the research been considered in the formulation of review recommendations	Were the specific directives for new research appropriate? in the discussion section of the report, of where future research direction should lie.	Quality
Castaño-Suarez 2024	YES	Unclear	YES	YES	YES	YES	YES	YES	YES	YES	YES	Good (8-11/11)
Lin et al. 2024	YES	YES	YES	YES	YES	YES	YES	YES	YES	YES	YES	Good (8-11/11)
Verma et al. 2023	YES	YES	YES	YES	YES	YES	YES	YES	YES	YES	YES	Good (8-11/11)
Larvin et al. 2023	NO	YES	YES	YES	YES	YES	YES	YES	YES	YES	YES	Good (8-11/11)
Le et al. 2022	NO	YES	YES	YES	YES	YES	YES	YES	YES	YES	YES	Good (8-11/11)
Yang et al. 2023 (COPD)	YES	YES	YES	YES	YES	YES	YES	YES	YES	YES	YES	Good (8-11/11)
Rosário-Dos-Santos et al. 2023	YES	YES	YES	YES	YES	YES	YES	YES	YES	YES	YES	Good (8-11/11)
Dewan et al. 2024	NO	YES	YES	YES	YES	YES	Unclear	YES	NO	YES	YES	Good (8-11/11)
Mirzaei et al. 2021	NO	Unclear	YES	YES	YES	YES	YES	YES	YES	YES	YES	Good (8-11/11)
Zheng et al. 2021	NO	YES	YES	YES	YES	YES	YES	YES	YES	YES	YES	Good (8-11/11)
Yang et al. 2023 (SJOGREN)	YES	Unclear	YES	YES	YES	YES	YES	YES	YES	YES	YES	Good (8-11/11)
Tan et al. 2024	NO	YES	YES	YES	YES	YES	YES	YES	YES	YES	YES	Good (8-11/11)
Leelaviwat et al. 2023	NO	YES	Unclear	YES	NO	NO	NO	Unclear	NO	NO	YES	Low (0-3/11)
Leng et al. 2023	YES	YES	YES	YES	YES	YES	YES	YES	YES	YES	YES	Good (8-11/11)
Ma et al. 2024	YES	YES	YES	YES	YES	YES	YES	YES	YES	YES	YES	Good (8-11/11)
Aguiar et al. 2024	NO	YES	YES	YES	YES	YES	YES	YES	YES	YES	YES	Good (8-11/11)
Xu et al. 2022	NO	YES	YES	YES	YES	YES	YES	YES	YES	YES	YES	Good (8-11/11)
Li et al. 2022	YES	Unclear	YES	YES	YES	YES	YES	YES	YES	YES	YES	Good (8-11/11)
Leelaviwat et al. 2024	NO	Unclear	YES	YES	NO	NO	NO	YES	NO	YES	YES	Medium (4-7/11)
Li et al. 2021	YES	YES	YES	YES	YES	NO	YES	YES	YES	YES	YES	Good (8-11/11)
Kim et al. 2022	NO	Unclear	Unclear	YES	YES	YES	YES	YES	YES	YES	YES	Medium (4-7/11)

Supplementary material 4. JBI critical appraisal checklist for systematic reviews

Systematic review	Is the review question clearly and explicitly stated? P(I/E)CO question	Were the inclusion criteria appropriate for the review question?	Was the search strategy appropriate? A systematic review should provide evidence of the search strategy that has been used to locate the evidence. keywords, language, limits, years, etc.	Were the sources and resources used to search for studies adequate? At least 2 databases, grey literature, reference list, contacts	Were the criteria for appraising studies appropriate? Risk of bias o any tool to assess the quality	Was critical appraisal conducted by two or more reviewers independently	Were there methods to minimize errors in data extraction? Strategies to minimize bias may include conducting all data extraction in duplicate and independently, using specific tools or instruments to guide data extraction and some evidence of piloting or training around their use	Were the methods used to combine studies appropriate?	Was the likelihood of publication bias assessed? Explicit statement of publication bias assessed by funnel plot or eggers test	Were recommendations for policy and/or practice supported by the reported data? In the discussion: Is there evidence that the strength of the findings and the quality of the research been considered in the formulation of review recommendations	Were the specific directives for new research appropriate? in the discussion section of the report, of where future research direction should lie.	Quality
Wang et al. 2024	YES	YES	YES	YES	YES	YES	YES	YES	YES	YES	YES	Good (8-11/11)
Mirzaei et al. 2022	NO	Unclear	YES	YES	YES	YES	YES	YES	YES	YES	NO	Good (8-11/11)
Liu et al. 2023	YES	Unclear	YES	YES	YES	YES	YES	YES	YES	YES	YES	Good (8-11/11)
